# Multi-centre, three arm, randomized controlled trial on the use of methylprednisolone and unfractionated heparin in critically ill ventilated patients with pneumonia from SARS-CoV-2 infection: A structured summary of a study protocol for a randomised controlled trial

**DOI:** 10.1186/s13063-020-04645-z

**Published:** 2020-08-17

**Authors:** Stefano Busani, Martina Tosi, Pasquale Mighali, Paola Vandelli, Roberto D’Amico, Marco Marietta, Francesco Forfori, Abele Donati, Gilda Cinnella, Amato De Monte, Daniela Pasero, Giacomo Bellani, Carlo Tascini, Giuseppe Foti, Marco Ranieri, Massimo Girardis

**Affiliations:** 1 Anaesthesia and Intensive Care Unit, Terapia Intensiva Polivalente, Policlinico di Modena, Azienda Ospedaliero-Universitaria di Modena, Ospedale Policlinico, Via del Pozzo, 71, 41124 Modena, Italy; 2 Servizio Formazione, Ricerca e Innovazione, Azienda Ospedaliero-Universitaria di Modena, Ospedale Policlinico, Modena, Italy; 3 Cattedra di Statistica Medica, Dipartimento di Scienze Mediche e Chirurgiche Materno-Infantili e dell’Adulto, Università di Modena e Reggio Emilia, Modena, Italy; 4 Haematology Unit, Azienda Ospedaliero-Universitaria di Modena, Ospedale Policlinico, Modena, Italy; 5 Dipartimento di Anestesia e Terapia Intensiva, Azienda Ospedaliera Universitaria Pisana, Pisa, Italy; 6 Anaesthesia and Intensive Care Unit, Department of Biomedical Sciences and Public Health, Università Politecnica delle Marche, Torrette di Ancona, Italy; 7 Department of Anesthesia and Intensive Care, O.O. Riuniti Hospital, University of Foggia, Foggia, Italy; 8 Anesthesiology and Intensive Care 1, Department of Anesthesia and Intensive Care, Azienda Sanitaria Universitaria Integrata-Udine, Udine, Italy; 9 Department of Medical, Surgical and Experimental Science, University of Sassari, Sassari, Italy; 10 Department of Emergency and Intensive Care, San Gerardo Hospital, Monza, Italy; 11 Clinica di Malattie Infettive, Azienda Sanitaria Universitaria Friuli centrale, Udine, Italy; 12 Department of Emergency Medicine, San Gerardo Hospital, Monza, Italy; 13 Department of Medicine and Surgery, University of Milan-Bicocca, Monza, Italy; 14 Anesthesia and Intensive Care Medicine, University Hospital of Bologna Sant’Orsola, Alma Mater Studiorum, University of Bologna, Bologna, Italy

**Keywords:** COVID-19, Randomized controlled trial, Protocol, Low-molecular weight heparin, Unfractionated heparin, Methylprednisolone, Mechanical ventilation, SARS-CoV-2 Pneumonia

## Abstract

**Objectives:**

To assess the hypothesis that an adjunctive therapy with methylprednisolone and unfractionated heparin (UFH) or with methylprednisolone and low molecular weight heparin (LMWH) are more effective in reducing any-cause mortality in critically-ill ventilated patients with pneumonia from SARS-CoV-2 infection compared to LMWH alone.

**Trial design:**

The study is designed as a multi-centre, interventional, parallel group, superiority, randomized, investigator sponsored, three arms study. Patients, who satisfy all inclusion criteria and no exclusion criteria, will be randomly assigned to one of the three treatment groups in a ratio 1:1:1.

**Participants:**

Inpatients will be recruited from 8 Italian Academic and non-Academic Intensive Care Units

**Inclusion Criteria (all required):**

1. Positive SARS-CoV-2 diagnostic (on pharyngeal swab of deep airways material)

2. Positive pressure ventilation (either non-invasive or invasive) from > 24 hours

3. Invasive mechanical ventilation from < 96 hours

4. PaO_2_/FiO_2_ ratio lower than 150 mmHg

5. D-dimer level > 6 times the upper limit of normal reference range

6. C-reactive Protein > 6-fold upper the limit of normal reference range

**Exclusion Criteria:**

1. Age < 18 years

2. On-going treatment with anticoagulant drugs

3. Platelet count < 100.000/mm^3^

4. History of heparin-induced thrombocytopenia

5. Allergy to sodium enoxaparin or other LMWH, UFH or methylprednisolone

6. Active bleeding or on-going clinical condition deemed at high risk of bleeding contraindicating anticoagulant treatment

7. Recent (in the last 1 month prior to randomization) brain, spinal or ophthalmic surgery

8. Chronic assumption or oral corticosteroids

9. Pregnancy or breastfeeding or positive pregnancy test. In childbearing age women, before inclusion, a pregnancy test will be performed if not available

10. Clinical decision to withhold life-sustaining treatment or “too sick to benefit”

11. Presence of other severe diseases impairing life expectancy (e.g. patients are not expected to survive 28 days given their pre-existing medical condition)

12. Lack or withdrawal of informed consent

**Intervention and comparator:**

*• LMWH group*: patients in this group will be administered enoxaparin at standard prophylactic dosage.

*• LMWH + steroid group*: patients in this group will receive enoxaparin at standard prophylactic dosage and methylprednisolone.

*• UFH + steroid group*: patients in this group will receive UFH at therapeutic dosages and methylprednisolone.

UFH will be administered intravenously in *UFH + steroid group* at therapeutic doses. The infusion will be started at an infusion rate of 18 UI/kg/hour and then modified to obtain aPTT Ratio in between the range of 1.5-2.0. aPTT will be periodically checked at intervals no longer than 12 hours. The treatment with UFH will be administered up to ICU discharge. After ICU discharge anticoagulant therapy may be interrupted or switched to prophylaxis with LMWH in the destination ward up to clinical judgement of the attending physician.

Enoxaparin will be administered in both *LMWH group* and *LMWH + steroid group* at standard prophylactic dose (i.e., 4000 UI once day, increased to 6000 UI once day for patients weighting more than 90 kg). The treatment will be administered subcutaneously once a day up to ICU discharge. After ICU discharge it may be continued or interrupted in the destination ward up to clinical judgement of the attending physician.

Methylprednisolone will be administered in both *LMWH + steroid group* and *UHF + steroid group* intravenously with an initial bolus of 0,5 mg/kg followed by administration of 0,5 mg/kg 4 times daily for 7 days, 0,5 mg/kg 3 times daily from day 8 to day 10, 0,5 mg/kg 2 times daily at days 11 and 12 and 0,5 mg/kg once daily at days 13 and 14.

**Main Outcomes:**

Primary Efficacy Endpoint:

All-cause mortality at day 28

Secondary Efficacy Endpoints:

- Ventilation free days (VFDs) at day 28, defined as the total number of days that patient is alive and free of ventilation (either invasive or non-invasive) between randomization and day 28 (censored at hospital discharge).

- Need of rescue administration of high-dose steroids or immune-modulatory drugs;

- Occurrence of switch from non-invasive to invasive mechanical ventilation during ICU stay;

- Delay from start of non-invasive ventilation to switch to invasive ventilation;

- All-cause mortality at ICU discharge and hospital discharge;

- ICU free days (IFDs) at day 28, defined as the total number of days between ICU discharge and day 28.

- Occurrence of new infections from randomization to day 28; including infections by Candida, Aspergillus, Adenovirus, Herpes Virus e Cytomegalovirus

- Occurrence of new organ dysfunction and grade of dysfunction during ICU stay.

- Objectively confirmed venous thromboembolism, stroke or myocardial infarction;

Safety endpoints:

- Occurrence of major bleeding, defined as transfusion of 2 or more units of packed red blood cells in a day, bleeding that occurs in at least one of the following critical sites [intracranial, intra-spinal, intraocular (within the corpus of the eye; thus, a conjunctival bleed is not an intraocular bleed), pericardial, intra-articular, intramuscular with compartment syndrome, or retroperitoneal], bleeding that necessitates surgical intervention and bleeding that is fatal (defined as a bleeding event that was the primary cause of death or contributed directly to death);

- Occurrence of clinically relevant non-major bleeding, defined ad acute clinically overt bleeding that does not meet the criteria for major and consists of any bleeding compromising hemodynamic; spontaneous hematoma larger than 25 cm^2^, intramuscular hematoma documented by ultrasonography, haematuria that was macroscopic and was spontaneous or lasted for more than 24 hours after invasive procedures; haemoptysis, hematemesis or spontaneous rectal bleeding requiring endoscopy or other medical intervention or any other bleeding requiring temporary cessation of a study drug.

**Randomization:**

A block randomisation will be used with variable block sizes (block size 4-6-8), stratified by 3 factors: Centre, BMI (<30/≥30) and Age (<75/≥75). Central randomisation will be performed using a secure, web-based, randomisation system with an allocation ratio of 1:1:1. The allocation sequence will be generated by the study statistician using computer generated random numbers.

**Blinding (masking):**

Participants to the study will be blinded to group assignment.

**Numbers to be randomised (sample size):**

The target sample size is based on the hypothesis that the combined use of UHF and steroid versus the LMWH group will significantly reduce the risk of death at day 28. The overall sample size in this study is expected to be 210 with a randomization 1:1:1 and seventy patients in each group. Assuming an alpha of 2.5% (two tailed) and mortality rate in LMWH group of 50%, as indicated from initial studies of ICU patients, the study will have an 80% power to detect at least a 25 % absolute reduction in the risk of death between: a) LMHW + steroid group and LMWH group or b) UHF + steroid group and LMWH group. The study has not been sized to assess the difference between LMHW + steroid group and UHF + steroid group, therefore the results obtained from this comparison will need to be interpreted with caution and will need further adequately sized studies confirm the effect.

On the basis of a conservative estimation, that 8 participating sites admit an average of 3 eligible patients per month per centre (24 patients/month). Assuming that 80 % of eligible patients are enrolled, recruitment of 210 participants will be completed in approximately 10 months.

**Trial Status:**

Protocol version 1.1 of April 26^th^, 2020.

Recruitment start (expected): September 1^st^, 2020

Recruitment finish (expected): June 30^th^, 2021

**Trial registration:**

EudraCT number 2020-001921-30, registered on April 15^th^, 2020

AIFA approval on May 4^th^, 2020

**Full protocol:**

The full protocol is attached as an additional file, accessible from the Trials website (Additional file 1). In the interest in expediting dissemination of this material, the familiar formatting has been eliminated; this Letter serves as a summary of the key elements of the full protocol.

## Supplementary information

**Additional file 1.**

## Data Availability

The study data will be collected during the entire study period in a dedicated electronic Case Report Form (eCRF) provided by the Steering Committee (SC). Data will be collected and stored on the hospital server, which will be protected by password to prevent unintentional modification or deletion.

