# Supplementary material for: Multi-centre, three arm, randomized controlled trial on the use of methylprednisolone and unfractionated heparin in critically ill ventilated patients with pneumonia from SARS-CoV-2 infection: A structured summary of a study protocol for a randomised controlled trial
Source: Trials. 2020 Aug 17;21:724. doi: 10.1186/s13063-020-04645-z (PMC7429933; doi:10.1186/s13063-020-04645-z)
Supplement: Supplementary file 1 — Additional file 1. [file 13063_2020_4645_MOESM1_ESM.pdf]

CLINICAL STUDY PROTOCOL

**Study Title:**	**Steroids and unfractionated heparin in critically ill patients with pneumonia from COVID-19 infection. A multicenter, interventional, randomized, three arms study design.**
**Short title:**	**STAUNCH-19 (STeroids And UNfraCtionated Heparin in covid-19 patients)**
**EudraCT N°:**	2020-001921-30
**Sponsor:**	University Hospital of Modena, L.go del Pozzo, 71, 41100 Modena- Italy.
**Study Coordinator:**	Prof. Massimo Girardis, Intensive Care Unit, Department of Anaesthesiology and Intensive Care, University Hospital of Modena, L.go del Pozzo, 71, 41100 Modena- Italy.
**Financial Support:**	Azienda Ospedaliero-Universitaria di Modena
**Contact Information:**	Prof. Massimo Girardis, Intensive Care Unit, Department of Anaesthesiology and Intensive Care, University Hospital of Modena, Modena Italy.<br>Business address: Largo del Pozzo 71, 41125 Modena, Italy.<br>E-mail address: girardis.massimo@unimore.it<br>Telephone number: +39 059 4224896.
**Investigational Product**	Unfractionated Heparin and Methylprednisolone
**Clinical Study Phase:**	III
**Version:**	1.1
**Issue Date:**	26/Apr/2020

PROTOCOL SIGNATURE FORM

Protocol Title: Steroids and unfractionated heparin in critically ill patients with pneumonia from COVID-19 infection: a multicenter, interventional, randomized, three arms study design.

Version: 1.1

Version Date: 26-Apr-2020

I have read the protocol described below and agree to conduct this study in accordance with procedures described therein. I also agree to conduct the study in compliance with all applicable regulations.

____
Principal Investigator's printed name

____ Date _________________________
Signature

INDEX

Protocol Signature Form **2**

List of abbreviations **5**

Roles and responsibilities **6**

1.0 Introduction **7**

1.1 Background and rationale 7

1.1.1 Benefits on the use of steroids 7

1.1.2 Benefits on the use of unfractionated heparin 8

1.1.3 Rationale 8

1.2 Study objective 8

1.3 Trial design 9

2.0 Methods: participants, Interventctions and outcomes **9**

2.1 Study setting 9

2.2 Eligibility criteria 9

2.2.1 Inclusion Criteria 9

2.2.2 Exclusion criteria 9

2.3 Interventions 10

2.4 Outcomes 11

2.4.1 Primary Endpoint 11

2.4.2 Secondary Endpoints 11

2.4.3 Physiological and Processes of care outcomes 11

2.5 Participant timeline 12

2.6 Sample size 12

2.7 Recruiment 13

3.0 Methods:assignment of interventions **13**

3.1 Allocation and blinding 13

4.0 Methods: data collection, management and analysis **13**

4.1 Data collection and management 13

4.2 Statistical Methods 14

4.2.1 Methods for statistical analysis 14

5.0 Methods: monitoring **15**

5.1 Data monitoring 15

5.2 Harms 15

5.3 Auditing 16

6.0 Ethics and dissemination **16**

6.1 Research ethics approval 16

6.2 Consent and confidentiality 17

6.3 Declaration of interest 18

6.4 Dissemination policy 18

6.5 Bibliography 19

APPENDIX 1: List of centres 22

APPENDIX 2: Flow-chart of the study 23

APPENDIX 3: Sequential Organ Failure Assessment (SOFA) score 24

APPENDIX 4: Study timeline 25

LIST OF ABBREVIATIONS

AE:	Adverse event
ADR:	Adverse drug reaction
AIFA:	Italian medicines agency
aPTT:	Adjusted Partial Pro-thrombin time
ALT:	Alanine aminotransferase
ARDS:	Acute Respiratory distress syndrome
AST:	Aspartate aminotransferase
BGA:	blood gas analysis
CMV:	cytomegalovirus
COVID-19:	Corona Virus 19 Disease
CRP:	C-reactive protein
DIC:	Disseminated Intravascular coagulation
DSMB:	Data Safety Monitoring Board
EC:	ethical committee
eCRF:	electronic Case Report Form
GCP:	good clinical practice
H:	hours
Hb:	Haemoglobin
HCT:	Hematocrit
HFDs:	Hospital free days
IFDs:	Intensive Care Unit free days
IL-6	Interleukin-6
HR:	Hearth rate
ICU:	Intensive Care Unit
INR:	International normalised ratio
LMWH:	Low Molecular Weight Heparin
MAP:	Mean arterial pressure
MRC:	Medical Research Council
n-CoV:	New Corona Virus
PCT:	Procalcitonin
PI:	principal investigator
PLT:	platelets
PT:	pro-thrombin time
RR:	Respiratory rate
SAPS II:	Simplified Acute Physiology Score II
SC:	Steering Committee
SOFA::	Sequential Organ Failure Assessment
UFH:	Unfractionated Heparin
VFDs:	Ventilation free days
WHO:	World Health Organization

Roles and responsibilities:

Principal investigator and coordinating centre	Girardis Massimo	University of Modena and Reggio Emilia, Italy
Statistical design, data management and analysis	D'Amico Roberto	University of Modena and Reggio Emilia, Italy
Steering Committee	Marco Marietta	University Hospital of Modena
	Stefano Busani	University of Modena and Reggio Emilia, Italy
	Francesco Forfori	University Hospital of Pisa, Italy
	Donati Abele	University of Ancona, Italy
	Gilda Cinella	University of Foggia, Italy
	Amato De Monte	University Hospital of Udine, Italy
	Pasero Daniela	University of Sassari, Italy
	Giacomo Bellani	University of Milano Bicoccca
	Ranieri Marco	University of Bologna

This is an investigator-initiated study. The steering committee will take responsibility for study design and data analysis and will operate actions necessary to guarantee that the trial is conducted in accordance with procedures described in this document and good clinical practice. The funders will have no role in study design, data collection, management, analysis, data interpretation, manuscript writing, or in the decision to submit manuscripts for publication.

1.0 INTRODUCTION

1.1 BACKGROUND AND RATIONALE

In December 2019, a cluster of acute respiratory illness caused by SARS-CoV-2 virus occurred in Wuhan, Hubei Province, China. The disease has rapidly spread from Wuhan to many other countries worldwide, rapidly becoming a global health emergency.

Although most patients have mild manifestations and good prognosis after infection, some patients develop severe symptoms and die from multiple organ complications. Pneumonia is the most frequent and serious complication of COVID-19, the disease that results from SARS-CoV-2 infection.

The pathogenesis of SARS-CoV-2 infection in humans remains unclear, although it is very likely that the most severe manifestations of COVID-19 may be linked to host-pathogen interaction immune mechanisms.

SARS-CoV-2 infection seems to induce in most critical cases an excessive and aberrant hyper-inflammatory host immune response that is associated with a so-called "cytokine storm", characterized by plasma increase of infection-related biomarkers and of many cytokines and chemokines documented in many observational studies [1,2]. These immunological derangements result in acute respiratory distress syndrome, characterized by typical radiological findings and that produce long-term damages such as fibrosis of lung tissue and organ harms. Moreover, during hospitalization, most patients were shown to have other immunological disorders such as marked lymphopenia, which was more pronounced over time among non survivors and specific depletion of regulatory T-lymphocytes [1,3].

This hyper-inflammatory immune response model is frequently observed in most severe forms of community acquired pneumonia and ARDS, where the detection of a strong systemic cytokine response (quantified with interleukin-6 and -10) is linked to poor therapeutic response and greater mortality [4,5].

Pro-thrombotic derangements of haemostatic system is another common finding in most severe forms of COVID-19 infections, which may be explained by the activation of coagulative cascade primed by inflammatory stimuli, in line with what is observed in many other forms of sepsis [6]. As a matter of fact, D-dimer elevation and CID-score alteration are common finding in this patients and seems to correlate with patients' outcome [3,7].

Currently, while many antiviral and immune-modulatory drugs are under investigation, there is no proven specific antiviral drug against SARS-CoV-2 virus, the only treatment consist in support therapies such as mechanical ventilation. Targeting inflammatory responses along with thrombosis prevention may be a promising therapeutic option to improve patients' outcome.

1.1.2 Benefits on the use of steroids

Studies on community-acquired pneumonia show that the excessive production of locally pulmonary cytokines induced by pulmonary infection may cause a severe host inflammatory response inducing pulmonary dysfunction, increasing ICU admission and mortality [8,9]. Steroids exert potent anti-inflammatory activity by inhibition of leucocytes extravasation, function of macrophages and antigen-presenting cells, production of TNF-alpha, interleukin-1 and nitric oxide. A Chinese report on 201 patients with COVID-19 pneumonia pointed out a survival benefit of more than 15% among patients with ARDS who received Methylprednisolone compared to those who did not[2].

An interesting report on post-mortem pathological findings in a SARS-CoV-19 patient with ARDS found out interstitial mononuclear inflammatory infiltrates dominated by lymphocytes, in both lungs, implying a severe

immune injury, pulmonary oedema and hyaline membrane formation, finding that suggest a possible role of steroids in this disease' treatment[10].

Nonetheless, corticosteroid treatment is a double-edged sword and their use remains controversial.

World Health Organization (WHO) recommend against the systematic use of corticosteroids for treatment of viral pneumonia. This recommendation is mainly driven by retrospective observational studies led on SARS, MERS and influenza virus showing no survival benefit and a decelerated viral clearance without affecting the outcome [11] [12] [13] together with the consideration of the many steroid-related possible harms such as secondary infections, gastro-intestinal bleeding and long-terms side-effects.

On the other hand, WHO itself through the Blueprint process has prioritized studies on steroids as an additional therapy that can improve patients' outcome in order to identify actions that can save lives in the immediate aftermath of the COVID-19 pandemic.

Because of methodological limitations in the available evidence, some scientific societies such as Chinese Thoracic Society have developed an expert consensus statement sustaining the use of corticosteroids in 2019-nCoV pneumonia.

1.1.3 Benefits on the use of unfractionated heparin

Several studies have demonstrated the tight interconnection between thrombosis and inflammation [14] [15] , two processes mutually reinforcing. Both coagulation factors [16] [17] [18] and platelets [19] [20] [21] are directly implicated in the modulation of the host immune response, displaying pro-inflammatory functions independent of their haemostatic effects.

Very recently, a severe derangement in coagulation parameters, showing a pro-thrombotic tendency, has been associated to a poor prognosis in patients affected by COVID-19 [22]. Moreover, in the same patients'' cohort, the use of Low Molecular Weight Heparin (LMWH) or Unfractionated Heparin (UFH) at prophylactic doses has been demonstrated to be associated with a reduced 28-day in more severe patients with Sepsis Induced Coagulopathy (SIC) score ≥4 (40.0% vs 64.2%, P=0.029), or D-dimer > 6 fold of upper limit of normal (32.8% vs 52.4%, P=0.017) [23].

This finding agrees with the already described immune-modulatory properties and protective action of glycocalyx from shedding displayed by heparin [24].

Despite the biological plausibility, no good evidence is available on the efficacy and safety of heparin on sepsis patients, and many issues have to be addressed, regarding the proper timing, dosages and administration schedules of anticoagulant drugs [25] [26] [27].

1.2 STUDY OBJECTIVE

The primary objective is to assess the hypothesis that an adjunctive therapy with steroids and unfractionated heparin or with steroids and molecular weight heparin (LMWH) are more effective in reducing any-cause mortality in critically-ill patients with pneumonia from COVID- 19 infection compared to low molecular weight heparin (LMWH) alone. Mortality will be measured at 28 days.

A possible result showing the efficacy of the composite treatment in reducing the mortality rate among critically ill patients with pneumonia from COVID-19 infection will lead to a revision of the current clinical approach to this disease.

1.3 TRIAL DESIGN

The study is designed as a multicenter, national, interventional, randomized, investigator sponsored, three arms study. Patients, who satisfy all inclusion criteria and no exclusion criteria, will be randomly assigned to one of the three treatment groups in a ratio 1:1:1.

2.0 METHODS: PARTICIPANTS, INTERVENCTIONS AND OUTCOMES

2.1 STUDY SETTING

The study will involve 8 Italian Academic and non-Academic Intensive Care Units.
See list of centres in APPENDIX 1.

2.2 ELIGIBILITY CRITERIA

2.2.1 Inclusion Criteria

1. Positive SARS-CoV-2 diagnostic (on pharyngeal swab of deep airways material)
2. Positive pressure ventilation (either non-invasive or invasive) from > 24 hours
3. Invasive mechanical ventilation from < 96 hours
4. P/F ratio $\leq$ 150
5. D-dimer level $\geq$ 6 x upper limit of local reference range
6. PCR $\geq$ 6 fold upper limit of local reference range

2.2.2 Exclusion Criteria

1. Age <18 years
2. On-going treatment with anticoagulant drugs
3. Platelet count < 100.000/mmc
4. History of heparin-induced thrombocytopenia
5. Allergy to sodium enoxaparine or other LMWH, unfractionated heparin or metylprednisolone;
6. Active bleeding or on-going clinical condition deemed at high risk of bleeding contraindicating anticoagulant treatment
7. Recent (in the last 1 month prior to randomization) brain, spinal or ophthalmic surgery
8. Chronic assumption or oral corticosteroids
9. Pregnancy or breastfeeding or positive pregnancy test. In childbearing age women, before inclusion, a pregnancy test will be performed if not available;
10. Clinical decision to withhold life-sustaining treatment or "too sick to benefit";
11. Presence of other severe diseases impairing life expectancy (e.g. patients are not expected to survive 28 days given their pre-existing medical condition);
12. Lack or withdrawal of informed consent.

2.3 INTERVENTIONS

Patients who satisfy all inclusion criteria and no exclusion criteria will be randomly assigned to a LMWH group (Group 1), LMWH+steroids (group 2) or UFH+steroid group (Group 3).

The study in conceived as open-label: the patients and all the health-care personnel will be aware of the assigned group. The treatments will be initiated as soon as possible after randomization (maximum allowed starting time 12h after randomization). In both groups, before enrolment in the study it will be allowed the

administration of low-dosages steroids for ARDS treatment (maximum 320 mg of Metilprednisolone per day for a maximum of 2 days).

Group 1 (LMWH group): Patients in this group will be administered enoxaparin at standard prophylactic dose (i.e., 4000 UI once day, increased to 6000 UI once day for patients weighting more than 90 kg).

The treatment with enoxaparin will be initiated as soon as possible after randomization (maximum allowed starting time 12h after randomization). The treatment will be administered subcutaneously, daily up to ICU discharge. After ICU discharge it may be continued or interrupted in the destination ward up to clinical judgement of the attending physician.

Group 2 (LMWH + steroids group): Patients in this group will receive enoxaparin and methylprednisolone. Enoxaparin will be administered at standard prophylactic dose (i.e., 4000 UI once day, increased to 6000 UI once day for patients weighting more than 90 kg). The treatment will be administered subcutaneously daily up to ICU discharge. After ICU discharge it may be continued or interrupted in the destination ward up to clinical judgement of the attending physician. Methylprednisolone will be administered intravenously with an initial bolus of 0,5 mg/kg followed by administration of 0,5 mg/kg 4 times daily for 7 days, 0,5 mg/kg 3 times daily from day 8 to day 10, 0,5 mg/kg 2 times daily at days 11 and 12 and 0,5 mg/kg once daily at days 13 and 14.

Group 3 (UFH + steroid group): Patients in this group will receive unfractionated heparin and methylprednisolone. Unfractionated heparin will be administered intravenously at therapeutic doses. The infusion will be started at an infusion rate of 18 IU/kg/hour and then modified to attain APTT Ratio in the range 1.5-2.0. aPTT will be periodically checked at intervals no longer than 12 hours. The treatment with unfractionated heparin will be administered up to ICU discharge. After ICU discharge anticoagulant therapy may be interrupted or switched to prophylaxis with LMWH in the destination ward up to clinical judgement of the attending physician. Methylprednisolone will be administered intravenously with an initial bolus of 0,5 mg/kg followed by administration of 0,5 mg/kg 4 times daily for 7 days, 0,5 mg/kg 3 times daily from day 8 to day 10, 0,5 mg/kg 2 times daily at days 11 and 12 and 0,5 mg/kg once daily at days 13 and 14. The dosage and time schedule were defined accordingly with dosage used in the large trial on ARDS by Steinberg KP et al (33)

Concomitant medications:

Depending on their clinical status, patients will be treated according to the principles of the Good Clinical Practice and clinical judgement of the attending physician. No other pharmacological therapy or treatment will be influenced from the study protocol. There are no restrictions to concomitant treatments provided to patients in this study. All relevant concomitant medications and treatments taken or administered in the 24 hours before screening and during the study period will be recorded.

Upon clinical judgement of the attending physicians, the patients can receive rescue administration of high- dose steroids or immune-modulatory drugs. Need and timing of rescue treatments administration will be recorded.

Criteria for discontinuing or modifying allocated interventions:

The duration of study therapy will be until ICU discharge for LMWH and UFH and 7 days for Methylprednisolone. Patients may be prematurely discontinued from study protocol at the discretion of the Investigator, should any untoward effect occur (including an AE or clinically significant laboratory abnormality that, in the opinion of the Investigator, warrants the subject's permanent discontinuation of study protocol-directed care).

Strategies to improve adherence to protocols

The site PIs will take primary responsibility for training local staff and for ensuring that protocol compliance is achieved.

2.4 OUTCOMES

2.4.1 Primary Endpoint:

- All-cause mortality at day 28.

2.4.2 Secondary Endpoints:

- Ventilation free days (VFDs) at day 28, defined as the total number of days that patient is alive and free of ventilation between randomisation and day 28 (censored at hospital discharge). Ventilation is considered as positive pressure ventilation, either invasive or non-invasive. Periods of assisted breathing lasting less than 24 hours for surgical procedures will not count against the ventilation free days calculation;
- Need of rescue administration of high-dose steroids or immune-modulatory drugs;
- Occurrence of switch from non-invasive to invasive mechanical ventilation during ICU-stay;
- Delay from start of non-invasive ventilation to switch to invasive ventilation;
- All-cause mortality at ICU discharge and hospital discharge;
- ICU free days (IFDs) at day 28, defined as the total number of days between ICU discharge and day 28. If death occurs during the ICU stay before day 28 the ICU free days calculation will be 0. The ICU readmission before day 28 after randomization will be considered;
- Occurrence of new infections from randomization to day 28; including infections by Candida, Aspergillus, Adenovirus, Herpes Virus e Citomegalovirus
- Occurrence of new organ dysfunction and grade of dysfunction during ICU stay. Organ dysfunction is defined as a Sequential Organ Failure Assessment (SOFA) score[28] ≥3 occurring after randomization, grade of dysfunction is measured with SOFA score (see SOFA score in APPENDIX 4) daily from randomization to ICU discharge (censored at day 28);
- Objectively confirmed venous thromboembolism, stroke or myocardial infarction;
- Occurrence of major bleeding (safety end point), defined as transfusion of 2 or more units of packed red blood cells in a day, bleeding that occurs in at least one of the following critical sites [intracranial, intraspinal, intraocular (within the corpus of the eye; thus, a conjunctival bleed is not an intraocular bleed), pericardial, intra-articular, intramuscular with compartment syndrome, or retroperitoneal], bleeding that necessitates surgical intervention and bleeding that is fatal (defined as a bleeding event that was the primary cause of death or contributed directly to death);
- Occurrence of clinically relevant non-major bleeding (safety end point) defined ad acute clinically overt bleeding that does not meet the criteria for major and consists of any bleeding compromising hemodynamics; spontaneous hematoma larger than 25 cm2, or 100 cm2, intramuscular hematoma documented by ultrasonography, haematuria that was macroscopic and was spontaneous or lasted for more than 24 hours after invasive procedures; haemoptysis, hematemesis or spontaneous rectal bleeding requiring endoscopy or other medical intervention or any other bleeding requiring temporary cessation of a study drug.

2.4.3 Physiological and Processes of care outcomes

- Vital signs such as mean arterial pressure (MAP), hearth rate (HR), respiratory rate (RR), diuresis and systemic body temperature and fluid balance will be recorded daily from inclusion until ICU discharge (censored day 28);
- Routine laboratory test parameters for organ function assessment: haemoglobin, platelets count, white blood cells count, troponin, coagulative parameters (INR, PT, aPTT), parameters for liver (AST, ALT, bilirubin) and renal function (creatinine) will be recorded daily from inclusion to ICU discharge (censored at day 28);
- Blood cells count, C-reactive protein (CRP), procalcitonin (PCT) and interleukin 6 (IL-6) will be recorded daily from inclusion to ICU discharge (censored at day 28);
- Ventilation mode, inspired oxygen fraction and arterial blood gas analysis parameters will be recorded daily from inclusion to ICU discharge from (censored at day 28);
- Adjunctive treatment such as pronation cycles, Nitric Oxide or ECMO will be recorded daily from inclusion to ICU discharge from (censored at day 28);
- New blood, respiratory and urinary-tract infections will be recorded from randomization to day 28;
- Viral reactivation measured by CMV DNA titres will be recorded from randomization to day 28;
- Need of new renal replacement therapy (intermittent haemodialysis or continuous veno-venous hemofiltration) from randomization to 28 days.

2.5 PARTICIPANT TIMELINE

See APPENDIX 4 for a timeline-scheme of the trial.

2.6 SAMPLE SIZE

The target sample size is based on the hypothesis that the combined use of UHF and steroids versus the LMWH group will significantly reduce the risk of 28-day death. The overall sample size in this study is expected to be 210 with a randomization 1:1:1. Assuming an alpha of 2.5% (two tailed) and mortality rate in LMWH group of 50%, as indicated from initial studies of ICU patients, the study will have an 80% power to detect at least a 25 % absolute reduction in the risk of death between:

a) LMHW + steroids group and LMWH group

or

b) UHF + steroids group and LMWH group

The study has not been sized to assess the difference between LMHW + steroids group and UHF + steroids group, therefore the results obtained from this comparison will need to be interpreted with caution and will need further adequately sized studies to be confirmed.

2.7 RECRUIMENT

All centres participating in this study are experienced in investigator-initiated studies. On the basis of a conservative estimation, that 8 participating sites admit an average of 3 eligible patients per month per centre (24 patients/month). Assuming that 80 % of eligible patients are enrolled, recruitment of 210 participants will be completed in around 10 months. Every month, recruitment status will be evaluated, and a newsletter will be disseminated, including any practical, clinical or scientific issue arisen.

3.0 METHODS: ASSIGNEMENT OF INTERVENTIONS

3.1 Allocation and blinding

A block randomisation will be used with variable block sizes (block size 4-6-8), stratified by 3 factors: Centre, BMI (<30; ≥30) and Age (<75 e ≥75). Central randomisation will be performed using a secure, web-based, randomisation system. The allocation sequence will be generated by the study statistician using computer generated random numbers. The attending physician, according to the protocol and the randomization, will note the inclusion of the patient in the study. Clinicians will receive all the instructions to attend the protocol and paper printed instructions will be distributed in the participating centres. The local coordinator will be responsible of the training and information of the personnel about the study protocol. The study is conceived as open-label: the patients and all the healthcare personnel will be aware of the group allocation.

4.0 METHODS: DATA COLLECTION, MANAGEMENT AND ANALYSIS

4.1 Data collection and management

Every patient who meets the inclusion criteria will be included and randomised in the three groups. The study data will be collected along the entire study period in a dedicated electronic Case Report Form (eCRF). The eCRF will be provided by the steering committee with proper options to minimize data entry errors: the datasheet will incorporate un-amendable fixed intervals of values (for continuous variables) and pre-defined coding system (for binary or categorical variables). Data entry will be performed and double-checked from a dedicated researcher in each centre; in order to limit collection errors, 10% of all records will be randomly re- checked from the PI in each participating centre. The data collection will be also checked by a Clinical Monitor, by phone calls as agreed with the investigators. The study Monitor will be responsible for performing the monitoring in accordance with good clinical practice (GCP) guidelines. The investigators will agree on monitoring phone calls to assess the progress of the study, verify adherence to the protocol, check the eligibility of patients, the accuracy and completeness of the eCRF, check the correlation between the data reported into eCRF and those recorded in the hospital documents (medical records, patient registries, etc.), check for the correct reporting of adverse events, verify that evaluations planned and documentation of the study are properly stored and handled. Data will be collected and stored in on hardware supports in every participant centre and sent to the coordinating centre at the end of the study and protected by password to prevent unintentional modifications or deletion. Each satellite centre will monthly communicate and report via e-mail with the coordinating centre about number of recruited patients, eventual missing data or missing visit or any kind of problem correlated to data collection. Data related to the study will be stored for eventual further analysis or study purpose for 10 years after the end of the study. All the data about the included patients will be extrapolated from the clinical documentation and recorded in an eCRF from adequately trained researcher. Demographic information (gender, age) and co-morbidities, will be registered at the inclusion, severity of critical illness (quantified by the Simplified Acute Physiology Score II,

SAPS II) will be calculated by the data from the first 24 h of ICU stay. During the study duration clinical and laboratory parameters will be evaluated and recorded following the timeline schedule in APPENDIX 4: SOFA score and its components, vital signs such as mean arterial pressure (MAP), hearth rate (HR), respiratory rate (RR), systemic temperature, diuresis, fluid balance, data from routine laboratory test such as haemoglobin, platelets count, white blood cells count, troponin, coagulative parameters (INR, PT, aPTT), parameters for liver and renal function (AST, ALT, bilirubin, creatinine), oxygen inspired fraction, blood gas analysis results (BGA), ventilation mode, need of adjunctive therapy for ARDS (i.e. prone position, Nitric oxide, ECMO), blood cells count, C-reactive protein (PCR), procalcitonin (PCT), interleukin-6 (IL-6), immunoglobulin titers (IgG, IgM, IgA) , occurrence of ICU-acquired (from 48 hours after ICU admission) blood, respiratory and urinary-tract infection and the implicated microorganisms, reactivation of viral infections (CMV-DNA titres). Other recorded parameters will include duration and mode of ventilatory support in days, duration and type of antiviral, antibiotic and antifungal therapy in days, need of vasoactive drugs, use of immune-modulatory drugs or other adjunctive treatment for COVID-19 pneumonia.

As a blood-bank for possible further biochemical investigations (e.g. cytokines titers, different biomarkers), for each patient who will be included in the study a blood sample of about 6 ml will be collected at baseline, day 7 and 28 or at ICU discharge and stored, after centrifugation, at -70°C in the local laboratory of each site.

4.2 STATISTICAL METHODS

4.2.1 Methods for statistical analysis

All patients enrolled in the study will be entered in the full analysis set independently of his/her treatment time. The intention to treat population will be considered for primary analysis. A descriptive statistical analysis will be performed to describe every relevant variable. In general, categorical data will be presented using counts and percentages, whilst continuous variables will be presented using the number of patients, mean, standard deviation, median, minimum, and maximum. Denominators for calculation of percentages will be taken as the number of non-missing responses in the specified analysis population and treatment group unless otherwise stated, and percentages will be rounded to one decimal. Minima and maxima will usually be reported to the same level of accuracy as the raw data; means, medians and standard deviations will be presented to one further decimal place; standard errors (if presented) will be presented to 2 decimal places more than the raw data. 97.5 % confidence interval will be calculated for primary outcome and for the relevant secondary ones. All data recorded in the CRF will be listed. Distribution of SAPSII and SOFA score (total and for single organ) at admission, P/F ratio distribution at baseline and at day 3, biomarkers PCR, PCT and IL-6 P/F ratio distribution at baseline and at day 3 will be summarized.

The comparison between

1. LMHW + steroids group and LMWH group
2. UHF + steroids group and LMWH group

with binary outcomes will be performed by using Relative Risks (RRs), whereas for continuous data, the difference of means (DMs) will be used. Comparisons involving time to event data will be displayed by using Kaplan-Maier survival curves and summarised by Hazard Ratios (HRs).

All measures of association will be presented with their confidence intervals. A result will be considered as statistically significant if its p-values will be less than 0.025 (2.5%).

The analyses will be performed by using STATA software.

As the study has not been sized to assess the difference between LMHW + steroids group and UHF + steroids group, the results obtained from this comparison will be reported and will be interpreted with caution. The main analyses will not take into account the factors of stratification.

5.0 METHODS: MONITORING

5.1 Data monitoring

An independent Data Safety Monitoring Board (DSMB), consisting of 2 experts in clinical research in intensive care and 1bio-statistic will be established before patient enrolment. The DSMB Charter will be prepared by the steering committee and signed by the members of the DSMB before the trial commences. The DSMB will have access to all results and make the appropriate considerations about the appropriateness of the sample size, the efficiency and quality of data collection system, eventual occurrence of suspected protocol-related adverse event. The DSMB has the right to stop the trial for safety reasons.

Analyses ad interim

Considering a statistical approach based on two phases, an interim analysis is planned after the randomization of 90 patients (50% of sample size) for the double objective of monitoring safety and verifying the accuracy of the assumptions made for sample size estimation regarding the primary end-point event rate in relation to the anticipated survival benefit. With the interim analysis we will be able to evaluate whether there is a substantial superiority of one treatment. The obtained results will be evaluated by the DSMB and by the steering committee and, in case of significant differences in survival among the groups, all patients will be switched to the most promising treatment.

5.2 Harms

All the patients, regardless the inclusion in the study and the randomization group, will benefit from the best standard of care following the principles of Good Clinical Practice. Intensive care patients frequently develop life-threatening organ failure unrelated to study interventions and despite optimal management. Therefore, events that are part of the natural history of the primary disease process or expected complications of critical illness [32] will not be reported as adverse events (AE) in this study. All the included patients will be intensively monitored following the standard procedures of the Intensive Care and any suspected protocol-related AEs will be reported. The steering committee (SC) and the DSMB will evaluate the adverse events occurred during the study. For every AE, the SC and the DSMB must assess the intensity (severity) and causality (relationship to study treatment). Specifically, the intensity of events should be classified as mild, moderate, or severe. The assessment of causality will be based upon the categories of related and not related. These classifications should be based on the following definitions:

The intensity (severity) of a specific event is defined as follow:

Mild: AE which is easily tolerated;

Moderate: AE sufficiently discomforting to interfere with daily activity;

Severe: AE which prevents normal daily activities.

Causality assessment is required for all clinical investigation cases. The relationship between an AE and a product is considered:

Possibly related: if, after careful assessment, a possible connection with the use of the investigational product cannot be ruled out;

Not-related: if the AE is due to causes not related to the investigational product.

Events considered "Possible" related to the study treatment, if considered unexpected, will be reported to appropriate regulatory authorities.

All AEs which occur during the course of the study should be recorded within 24 hours in the eCRF. Information on the AE must be recorded on a specific AE form (included in the Investigator's Site File) and forwarded to the DSMB and to the safety board. Moreover, the eCRF will ensure that an automatic email will be generated and sent directly to the Principal Investigator accountable to receive the study safety information. Each Investigator will be informed of all AE which are reported from other Investigators periodically.

During the course of the clinical trial, the investigators shall report any serious unexpected suspected adverse event to the EC which approved the protocol and to the Competent Authorities as soon as possible and in no event later than:

(a) seven calendar days after becoming aware of the information if the event is fatal or life threatening; and

(b) fifteen calendar days after becoming aware of the information if the event is neither fatal nor life threatening.

The investigators will, within eight days after having informed the EC, submit a complete report in respect of that information that includes an assessment of the importance and implication of any findings made.

5.3 Auditing

The principal investigator and the SC will provide all the necessary information and material to the participating centres in order to standardize all the protocol-related procedures and to avoid unexpected variability between centres. A preliminary audit between the coordinating centre and the satellite centres will be performed before to start the recruitment phase to offer the major consensus and homogeneity possible. Printed and electronic informative material (complete original protocol, informed consent modules, informative modules for patients and relatives, recruitment check-list, graphic timeline of interventions and visits, order-list for physicians and nurses) will be distributed to every centre.

6.0 ETHICS AND DISSEMINATION

The study will be conducted in line with the protocol, the Declaration of Helsinki (1964) and subsequent amendments and updates (Fortaleza, Brazil, October 2013). Moreover, it is the responsibility of the investigator to ensure that the study will be done in line with to the requirements of Good Clinical Practice(GCP) and the applicable regulatory requirements.

6.1 Research ethics approval

The entire study protocol, including informative material for the patients and modules for the informed consent, will be evaluated by the Local Ethics Committee from the coordinating centre and from all the collaborating centres. The study will not start before obtaining a favourable opinion from the EC, the Competent Authority Authorization and any other authorization required by local regulation. Every intention to modify any element of the original protocol after the first approval will be promptly notified to the Ethics Committee and will be applied only after its written authorization.

Investigator/sponsor will be responsible to submit to the Ethics Committee any amendments to the protocol.

6.2 Consent and confidentiality

Before inclusion in the study, conscious patients must be informed of the purpose and of clinical procedures required by the protocol. The investigators will explain the purpose, risks and benefits associated with study participation. In addition, patients will be informed of his right to withdraw from the study at any time without explanation and without losing the right to future medical care.

If the patient will be unable to comprehend or to give his consent (because of compromised neurological status), the following consent options are acceptable: (i) A priori consent by a legal representative (ii) delayed consent from a legal representative; (iii) Delayed consent from the patient; (iv) waiver of consent; (v) consent provided by an ethics committee or other legal authority. Which options are available at individual participating sites will be determined by the relevant ethics committee and subject to applicable laws. In Italy, the approach to patients unable to provide an informed consent before enrolment will be to consider whether participation is in the best interests of each individual patient and as soon as it is practical and reasonable to do so, to seek the advice of persons interested in the patient's welfare (e.g. family member) to establish that study participation is consistent with the patient's wishes. It is important to know that the randomization does not preclude supportive therapy with IgM preparation but it may only modify the dosage strategies without any expected significant adverse effect for the patient. All participants who recover sufficiently will be given the opportunity to provide informed consent for ongoing study participation and for the use of data collected for the study. Every patient is free to leave the study protocol at any state of the study and can request to retire his consent and, consequently, to ask the elimination of all his data from the database.

In order to comply with legal requirements regarding privacy and the processing of sensitive personal data, Legislative Decree 30/06/2003 n. 196 on the protection of personal data and Regulation (EU) 2016/679 of the European Parliament and of the Council of 27 April 2016 on the protection of natural persons with regard to the processing of personal data and on the free movement of such data, and repealing Directive 95/46/EC (General Data Protection Regulation), each patient will be given an information sheet on the study they participate in and will be asked for sign the consent to the processing of personal data.

Data about personal and private information, included sensible data, will be treated following current legislation on data protection; patients will be identified with a coding system and data registered in anonymous form. Collected data will be processed by the Investigator for the exclusive purposes of fulfil to the present study requirements, and in anonymous form, aggregate in the study database with data obtained from the other patients participating, solely on the basis of finalizing the study and achievement objectives.

Obtained data will not be disclosed except in strictly anonymous and aggregated form. Direct access to the original medical records may be requested only by commissioning DMC of the study and will be accessible by a representative of the CRO, its delegate to perform monitoring on the conduct of the trial, the EC or by the regulatory Authorities, such as personnel of the Italian Ministry of Health and the Italian Medicines Agency (AIFA) to verify that the information entered in the documents of the study are correct and methods that guarantee the privacy and confidentiality of the data are respected. Such verification activities will be always carried out under the supervision of the SC, executed professionally and to guarantee the privacy of the subject.

6.3 Declaration of interest

The study participants declare no financial and/or other conflicts of interest related to the study.

6.4 Dissemination policy:

The Circ. Min. Health N° 6 of 09/02/2002 obliges each researcher who gets any results of interest to public health, to publish the results within 12 months from the end of the study. All the patients will freely agree or disagree to participate to the study in the belief that the results will be useful to improve knowledge about their pathologies, for health benefit from themselves or other patients. To respect their will and in the maximum interest of honest clinical research, the investigators agree on the need to ensure the wide publication and diffusion of their results in a consistent and responsible way under their responsibility. The study coordinator is the official data owner. The steering committee has the right to present methods and results of the study at public symposia and conferences. The principal publications from the trial will be in the name of Investigators with full credit assigned to all collaborating investigators and institutions.

6.5 BIBLIOGRAPHY

1. Qin, C. *e t al.* Dysregulation of immune response in patients with COVID-19 in Wuhan, China. *Clin. Infect. Dis. Off. Publ. Infect. Dis. Soc. Am.* (2020) doi:10.1093/cid/ciaa248.
2. Wu, C. *et al.* Risk Factors Associated With Acute Respiratory Distress Syndrome and Death in Patients With Coronavirus Disease 2019 Pneumonia in Wuhan, China. *JAMA Intern. Med.* (2020) doi:10.1001/jamainternmed.2020.0994.
3. Wang, D. *et al.* Clinical Characteristics of 138 Hospitalized Patients With 2019 Novel Coronavirus–Infected Pneumonia in Wuhan, China. *JAMA* **323**, 1061–1069 (2020).
4. Kellum, J. A. *et al.* Understanding the Inflammatory Cytokine Response in Pneumonia and Sepsis. *Arch. Intern. Med.* **167**, 1655–1663 (2007).
5. Calfee, C. S. *et al.* Subphenotypes in acute respiratory distress syndrome: latent class analysis of data from two randomised controlled trials. *Lancet Respir. Med.* **2**, 611–620 (2014).
6. Voves, C., Wuillemin, W. A. & Zeerleder, S. International Society on Thrombosis and Haemostasis score for overt disseminated intravascular coagulation predicts organ dysfunction and fatality in sepsis patients. *Blood Coagul. Fibrinolysis Int. J. Haemost. Thromb.* **17**, 445–451 (2006).
7. Lillicrap, D. Disseminated intravascular coagulation in patients with 2019-nCoV pneumonia. *J. Thromb. Haemost. JTH* **18**, 786–787 (2020).
8. Hotchkiss, R. S. & Karl, I. E. The pathophysiology and treatment of sepsis. *N. Engl. J. Med.* **348**, 138–150 (2003).
9. Meijvis, S. C. A. *et al.* Dexamethasone and length of hospital stay in patients with community-acquired pneumonia: a randomised, double-blind, placebo-controlled trial. *Lancet Lond. Engl.* **377**, 2023–2030 (2011).
10. Xu, Z. *et al.* Pathological findings of COVID-19 associated with acute respiratory distress syndrome. *Lancet Respir. Med.* **8**, 420–422 (2020).
11. Stockman, L. J., Bellamy, R. & Garner, P. SARS: Systematic Review of Treatment Effects. *PLoS Med.* **3**, (2006).
12. Delaney, J. W. *et al.* The influence of corticosteroid treatment on the outcome ofinfluenza A(H1N1pdm09)-related critical illness. *Crit. Care Lond. Engl.* **20**, 75 (2016).
13. Arabi, Y. M. *et al.* Corticosteroid Therapy for Critically Ill Patients with Middle East Respiratory Syndrome. *Am. J. Respir. Crit. Care Med.* **197**, 757–767 (2018).
14. Jackson, S. P., Darbousset, R. & Schoenwaelder, S. M. Thromboinflammation: challenges of therapeutically targeting coagulation and other host defense mechanisms. *Blood* **133**, 906–918 (2019).
15. Iba, T. & Levy, J. H. Inflammation and thrombosis: roles of neutrophils, platelets and endothelial cells and their interactions in thrombus formation during sepsis. *J. Thromb. Haemost. JTH* **16**, 231–241 (2018).

16. Claushuis, T. a. M. *et al.* Thrombin contributes to protective immunity in pneumonia-derived sepsis via fibrin polymerization and platelet-neutrophil interactions. *J. Thromb. Haemost. JTH* **15**, 744–757 (2017).
17. Burzynski, L. C. *et al.* The Coagulation and Immune Systems Are Directly Linked through the Activation of Interleukin-1α by Thrombin. *Immunity* **50**, 1033-1042.e6 (2019).
18. Chen, J. *et al.* Coagulation factors VII, IX and X are effective antibacterial proteins against drug-resistant Gram-negative bacteria. *Cell Res.* **29**, 711–724 (2019).
19. de Stoppelaar, S. F., van 't Veer, C. & van der Poll, T. The role of platelets in sepsis. *Thromb. Haemost.* **112**, 666–677 (2014).
20. Vardon-Bounes, F. *et al.* Platelets Are Critical Key Players in Sepsis. *Int. J. Mol. Sci.* **20**, 3494 (2019).
21. Assinger, A., Schrottmaier, W. C., Salzmann, M. & Rayes, J. Platelets in Sepsis: An Update on Experimental Models and Clinical Data. *Front. Immunol.* **10**, 1687 (2019).
22. Tang, N., Li, D., Wang, X. & Sun, Z. Abnormal coagulation parameters are associated with poor prognosis in patients with novel coronavirus pneumonia. *J. Thromb. Haemost. JTH* **18**, 844–847 (2020).
23. Tang, N. *et al.* Anticoagulant treatment is associated with decreased mortality in severe coronavirus disease 2019 patients with coagulopathy. *J. Thromb. Haemost. JTH* (2020) doi:10.1111/jth.14817.
24. Li, X. & Ma, X. The role of heparin in sepsis: much more than just an anticoagulant. *Br. J. Haematol.* **179**, 389–398 (2017).
25. Zarychanski, R. *et al.* The efficacy and safety of heparin in patients with sepsis: a systematic review and metaanalysis. *Crit. Care Med.* **43**, 511–518 (2015).
26. Yamakawa, K. *et al.* Identifying Sepsis Populations Benefitting from Anticoagulant Therapy: A Prospective Cohort Study Incorporating a Restricted Cubic Spline Regression Model. *Thromb. Haemost.* **119**, 1740–1751 (2019).
27. Wiewel, M. A. *et al.* Chronic antiplatelet therapy is not associated with alterations in the presentation, outcome, or host response biomarkers during sepsis: a propensity-matched analysis. *Intensive Care Med.* **42**, 352–360 (2016).
28. Vincent, J. L. *et al.* The SOFA (Sepsis-related Organ Failure Assessment) score to describe organ dysfunction/failure. On behalf of the Working Group on Sepsis-Related Problems of the European Society of Intensive Care Medicine. *Intensive Care Med.* **22**, 707–710 (1996).
29. Bhatraju, P. K. *et al.* Covid-19 in Critically Ill Patients in the Seattle Region — Case Series. *N. Engl. J. Med.* **0**, null (2020).
30. Lefering, R., Goris, J. R., Nieuwenhoven, E. J. van & Neugebauer, E. Revision of the multiple organ failure score. *Langenbecks Arch. Surg.* **387**, 14–20 (2002).
31. Rhodes, A. *et al.* Surviving Sepsis Campaign: International Guidelines for Management of Sepsis and Septic Shock: 2016. *Intensive Care Med.* **43**, 304–377 (2017).
32. Cook, D., Lauzier, F., Rocha, M. G., Sayles, M. J. & Finfer, S. Serious adverse events in academic critical care research. *CMAJ Can. Med. Assoc. J.* **178**, 1181–1184 (2008).
33. Steinberg KP, Hudson LD, Goodman RB, et al. Efficacy and safety of corticosteroids for persistent acute respiratory distress syndrome. N Engl J Med.2006 Apr 20;354(16):1671-84.

APPENDIX 1: List of centres

1. University of Modena and Reggio Emilia, Italy
2. University Hospital of Pisa, Italy
3. University of Ancona, Italy
4. University of Foggia, Italy
5. University Hospital of Udine, Italy
6. University of Sassari, Italy
7. University of Milano Bicoccca
8. University of Bologna

APPENDIX 2: flow-chart of the study

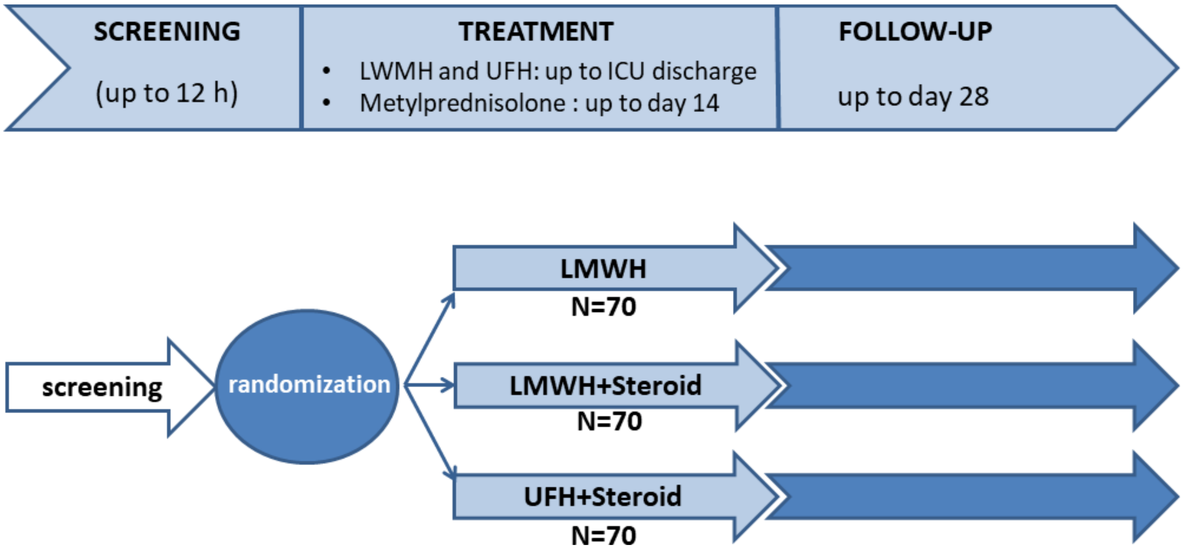

APPENDIX 3: Sequential Organ Failure Assessment (SOFA) score[1]

variables	0	1	2	3	4
**Central Nervous System (Glasgow Coma Scale score)**	15	13-14	10-12	6-9	<6
**Respiratory (PaO_2 / FiO_2 ratio)**	>400	≤ 400	≤ 300	≤ 200 [A]	≤ 200 [A]
**Cardiovascular, Hypotension (MAP; mmHg / catecholamine dose; γ/Kg/min)**	PAM >70 and NO cathecolamines	PAM < 70 and NO cathecolamines	Dop[B]≤ 5 or Dob[B] (any dose)	Dop[B]>5 or Epi[B]≤0,1 or Norepi[B]≤0,1	Dop[B]> 15 or Epi[B]>0,1 or Norepi[B]> 0,1
**Coagulation (Platelets ; x 10^3 u/µL)**	>150	≤ 150	≤ 100	≤ 50	≤ 20
**Liver (Bilirubin; mg/dl)**	<1,1	1,2 - 1,9	2,0 - 5,9	6,0 – 11,9	>12
**Renal (Creatinine; mg/dl /diuresis ;ml/24h)**	<1,1 + diuresis > 500	1,2 - 1,9 + diuresis > 500	2,0 - 3,4 + diuresis > 500	3,5 – 4,9 o diuresis <500	>5 o diuresis <200

A - Values are with respiratory support.
B – Cathecolamines are specified as follow:

Dop	Dopamine
Dob	Dobutamine
Epi	Epinephrine
Norepi	Norepinephrine

APPENDIX 4: Study timeline

Time-points		Day-0	From Day 0 to Say 28	Day-28	ICU discharge	Hospital discharge
**Eligibility Screen**[A]		X				
**Informed consent**		X<br>or as soon as feasible				
**Allocation**		X				
**Treatment:**	LMWH (group 1)	• Enoxaparine from day 0 to ICU discharge				
	LMWH + steroids (group 2)	• Enoxaparine: from day 0 to ICU discharge<br>• Methylprednisolone: from day 0 to day 14				
	UFH + steroids (group 3)	• Unfractionated heparin : from day 0 to ICU discharge<br>• Methylprednisolone: from day 0 to day 14				
**Baseline characteristics**[B]		X				
**Physiological and Processes of care outcomes**[C]			X	X		
**Safety monitoring**			X			
**Primary outcome**[D]				X		
**Secondary outcomes**[E]				X	X	X

A) Eligibility screen:
- Inclusion and exclusion criteria (see 2.2.1 and 2.2.2) including pregnancy test.

B) Baseline characteristics:
- Demographic data (sex, date of birth)
- Medical, Medication and surgical history
- SAPS II score

C) Physiological and Processes of care outcomes:
- Vital signs: mean arterial pressure (MAP), Hearth rate (HT), Respiratory rate (RR), diuresis and systemic body temperature and fluid balance will be recorded daily from inclusion to ICU discharge (censored day 28);
- Routine laboratory test parameters for organ function assessment:, haemoglobin, platelets count, white blood cells count, troponin, coagulative parameters (INR, PT, aPTT), parameters for liver (AST, ALT, bilirubin) and renal function (creatinine) will be recorded daily from inclusion to day 7 and then at day 14, 21, 28, censored at ICU discharge;
- Blood cells count, C-reactive protein (CRP) and procalcitonin (PCT), IL-6, immunoglobulins titers (IgG, IgA , IgM) will be recorded daily from inclusion to day 7 and then at day 14, 21 and 28, censored at ICU discharge;
- Beta-D Glucan in the blood and/or Galactomannan in the BAL will be recored at admission and then every 3-5 days, censored at ICU discharge
- Ventilation mode (spontaneous breathing or mechanical ventilation), inspired oxygen fraction and arterial blood gas analysis parameters will be recorded daily from inclusion to ICU discharge , censored at day 28;
  - Adjunctive treatment such as pronation cycles, Nitric Oxide or ECMO will be recorded daily from inclusion to ICU discharge from (censored at day 28);
- New blood, respiratory and urinary-tract infections and the implicated microorganisms will be recorded from randomization to day 28;
- Viral reactivation measured by ADENOVIRUS, HSV e CMV DNA titres will be recorded from randomization to day 28, censored at ICU discharge;
- Need of renal replacement therapy: from randomization to 28 days, censored at ICU discharge.

D) Primary Outcome:
- All-cause mortality will be measured at day 28.

E) Secondary Outcomes:
- All-cause mortality will be measured at ICU discharge and hospital discharge
- SOFA score and it's components will be recorded daily from randomization to ICU discharge (censored at day 28);
- Ventilation free days (VFDs) at day 28, measured at day 28;
- ICU free days (IFDs) at 28-day, measured at day 28;
- Need of rescue administration of high-dose steroids or immune-modulatory drugs, measured from randomization to ICU discharge, censored at day 28;
- Switch from non-invasive to invasive mechanical ventilation from randomization to ICU discharge, censored at day 28;
- Occurrence of new infections will be recorded from randomization to day 28;
- Occurrence of objectively confirmed venous thromboembolism, stroke or myocardial infarction will be recorded from randomization to day 28;
- Occurrence of protocol related adverse events (see chapter 5.2) at day 28 (safety endpoint).
